# Development and evaluation of a deep learning-assisted diagnostic support system for radiographer preliminary clinical evaluation of intracranial hemorrhage

**DOI:** 10.7717/peerj.21414

**Published:** 2026-06-17

**Authors:** Kazuma Tsukamoto, Mizuho Nishio, Ryo Kurosaki, Yasuyuki Kojita, Hidetoshi Matsuo, Munenobu Nogami, Kazuki Ishikawa, Ryosuke Hatano, Yuka Shimo, Yuki Komon, Yuya Ueki, Izumi Imaoka, Kazuyuki Ohmura, Kazuya Matsuo, Toshiaki Akashi, Shigeki Aoki, Akiko Kusaka, Takamichi Murakami, Yoshihiro Muragaki

**Affiliations:** 1 Department of Medical Device Engineering, Kobe University Graduate School of Medicine, Kusunoki-cho, Chuo-ku, Kobe, Japan; 2 Center for Radiology and Radiation Oncology, Kobe University Hospital, Kusunoki-cho, Chuo-ku, Kobe, Japan; 3 Department of Radiology, Kobe University Graduate School of Medicine, Chuo-ku, Kobe, Japan; 4 Division of Medical Imaging, Biomedical Imaging Research Center, University of Fukui, Yoshida, Fukui, Japan; 5 GE HealthCare (Japan) Research Division, Hino-shi, Tokyo, Japan; 6 Department of Radiology, Juntendo University Graduate School of Medicine, Bunkyo-ku, Tokyo, Japan

**Keywords:** Artificial intelligence, Deep learning, Intracranial hemorrhage, Computed tomography, Performance evaluation

## Abstract

**Background:**

Intracranial hemorrhage is life-threatening and requires prompt and accurate diagnosis. Non-contrast head computed tomography is the standard first-line examination, but detecting small hemorrhages and classifying multiple subtypes require substantial expertise. Workforce shortages and increasing diagnostic workloads, especially in emergency settings, further challenge timely decision-making. Artificial intelligence (AI)-assisted interpretation has shown promise for improving accuracy and efficiency. This retrospective study evaluated the effect of AI assistance on the diagnostic performance of radiologic technologists (RTs).

**Methods:**

We analyzed the data for 100 non-contrast head computed tomography examinations (50 positive and 50 negative for hemorrhage) obtained from the Japan Medical Image Database. The interpretations of the five RTs (5–12 years of experience) with and without AI assistance were compared with those of two radiologists. The detection targets were intraparenchymal, intraventricular, subarachnoid, subdural, epidural, and any hemorrhages. We calculated the Area Under the Receiver Operating Characteristic Curve (AUC), accuracy, sensitivity, and specificity. The differences in the AUC for the AI-assisted and unassisted readings were tested using the DeLong method with Bonferroni correction.

**Results:**

Significant AUC improvements were observed for five of the 30 reader–task comparisons (17%) after Bonferroni correction. These improvements were all related to intraventricular (*p* = 0.0001 to 0.0071) and subdural (*p* = 0.0022 to 0.0071) hemorrhages.

**Conclusion:**

AI assistance significantly improved RT detection of challenging subtypes such as intraventricular and subdural hemorrhages. However, it did not improve the diagnostic accuracy for detecting any hemorrhage overall (*p* = 0.0689 to 0.9669). AI can strengthen the role of RTs within task-sharing models and help stabilize preliminary assessments, especially in emergency care and resource-constrained environments.

## Introduction

Intracranial hemorrhage is life-threatening, and it requires prompt and accurate diagnosis (Caceres & Goldstein, 2012). Appropriate treatment within 24 h of symptom onset significantly improves clinical outcomes (van Asch et al., 2010). Non-contrast head computed tomography is the primary imaging modality for intracranial hemorrhage, but its interpretation for detecting small hemorrhage and classifying multiple hemorrhage patterns requires considerable expertise (Chan, 2007). These challenges are compounded by radiologist shortages and increasing interpretive workload, especially in emergency settings where expedited diagnosis is essential (Chan, 2007).

Deep learning–based automated analysis has advanced rapidly (Saba et al., 2019) and demonstrated promising outcomes for detecting intracranial hemorrhage and classifying its subtypes (Chilamkurthy et al., 2018). Convolutional neural networks (CNNs) have demonstrated high diagnostic accuracy for intracranial hemorrhage (Kuo et al., 2019), highlighting the potential value of artificial intelligence (AI)-assisted interpretation. AI has been reported to improve diagnostic accuracy and reduce interpretation time when deployed as an assistive tool for clinicians, and some studies have reported performance comparable to that of radiologists (Kuo et al., 2019; Kim et al., 2025).

Several countries, including those in Europe, have implemented task-sharing models in which radiologic technologists (RTs) provide Preliminary Clinical Evaluation or Preliminary Image Evaluation for acute imaging findings (Anudjo, Docherty & Akudjedu, 2025). These models have been associated with improved service continuity and reduced physician workload (Society and College of Radiographers, 2024; Hardy & Snaith, 2015). However, concrete strategies to enhance the interpretive performance of RTs have not been sufficiently explored (Alexander-Bates et al., 2021).

This study aimed to evaluate the extent to which an AI-based decision-support system can enhance the diagnostic performance of RTs for intracranial hemorrhage. The findings of this study will ascertain the effectiveness of AI-assisted imaging interpretation by RTs and provide foundational evidence to inform its practical implementation in clinical workflows.

## Materials and Methods

### Study design and ethical approval

This retrospective study used anonymized non-contrast head computed tomography (NCCT) data collected from the sites participating in the Japan Medical Image Database (JMID). It was approved by the Institutional Review Board of Kobe University Hospital (approval number B220230, approval date 2023/03/28) and the JMID project, and the requirement for individual informed consent was waived.

### Data source and case selection

**Figure 1:**
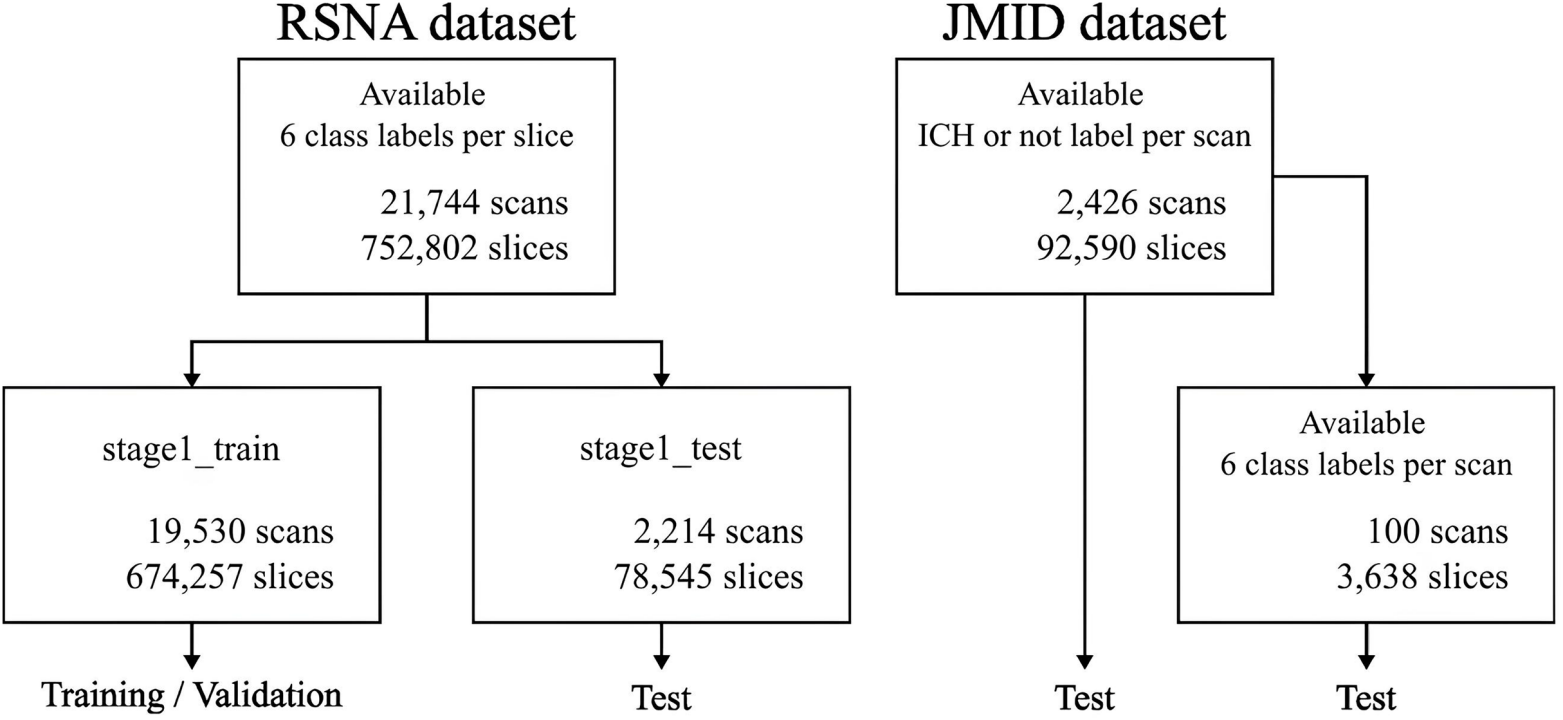
Two datasets used in this study.

JMID is a nationwide repository of clinical imaging data contributed by collaborating institutions across Japan. It was established with support from the Japan Agency for Medical Research and Development. JMID provides a robust foundation for developing and evaluating AI-based decision support systems. For the AI evaluation, we used data from 2,227 and 199 cases with and without intracranial hemorrhage (total, *n* = 2,426) from JMID, respectively. The presence or absence of intracranial hemorrhage in these 2,426 cases was determined based on radiologist interpretations, which served as the reference standard. In addition, 100 cases (50 positive and 50 negative) were sampled from the 2,426 cases. In these 100 cases, intracranial hemorrhage (ANY) and the subtypes—intraparenchymal hemorrhage (ICH), intraventricular hemorrhage (IVH), subarachnoid hemorrhage (SAH), subdural hemorrhage (SDH), and epidural hemorrhage (EDH)—were confirmed by a board-certified radiologist. These cases were used for the reader study and for evaluating the computer-aided system with AI. Tables 1 and 2 summarize the data for the 100 cases. The right panel of Fig. 1 outlines the JMID dataset size and usage.

**Table 1:** Patient demographics.

Parameter	Subjects (*n* = 100)
**Age**	
Mean	62.7 ± 22.1 years
Range	0‒97
**Sex**	
Men	55
Women	45

**Table 2:** Distribution of intracranial hemorrhage subtypes.

Subtypes	Subjects (*n* = 100)
Intraparenchymal hemorrhage (ICH)	24
Intraventricular hemorrhage (IVH)	20
Subarachnoid hemorrhage (SAH)	22
Subdural hemorrhage (SDH)	17
Epidural hemorrhage (EDH)	10
At least one subtype positive (ANY)	50

### AI system

We implemented a 2.5-D CNN-based classifier to detect and subtype hemorrhage on NCCT, following the approach of Wang et al. (2021), Scu-sen (2020). The Radiological Society of North America (RSNA) dataset stores each computed tomography (CT) slice in DICOM format and provides slice-level labels for hemorrhage (Radiological Society of North America, 2019). We simulated clinical interpretation by applying three window settings (brain, subdural, and bone) to each slice (Wang et al., 2021). The single-channel slice was converted into an 8-bit three-channel image based on these windows, and non-anatomical objects (*e.g*., CT scanner table) were removed. The target slice and the slices immediately superior and inferior to it were extracted from each CT volume to create a 3-slice stack. This was combined with the three windows to produce a 9-channel input (3 slices × 3 windows). The backbone network was an SE-ResNeXt-50 pretrained on ImageNet, and we fine-tuned it for our task (Deng et al., 2009; He et al., 2016; Hu, Shen & Sun, 2018). The results of five-fold cross-validation were averaged and used as the final prediction. The optimization was performed using Adam with a cosine annealing learning rate schedule (Loshchilov & Hutter, 2017). Data augmentation included horizontal flipping, coarse dropout, rotation, and shift-scale-rotation (Shorten & Khoshgoftaar, 2019). The system was implemented in PyTorch. The left panel of Fig. 1 summarizes the size of the RSNA dataset and its utilization.

### Readers and study protocol

Five RTs with 5, 6, 9, 10, and 12 years of experience and two radiologists with 18 and 8 years of experience, respectively, participated. The 100 cases were interpreted in three scenarios: (a) the RTs read the images and determined the presence and subtype of hemorrhage (RT without AI); (b) the RTs read the scans with access to the AI predictions and performed the same assessments (RT with AI); and (c) the radiologists read the images without AI and performed the same assessments.

**Figure 2:**
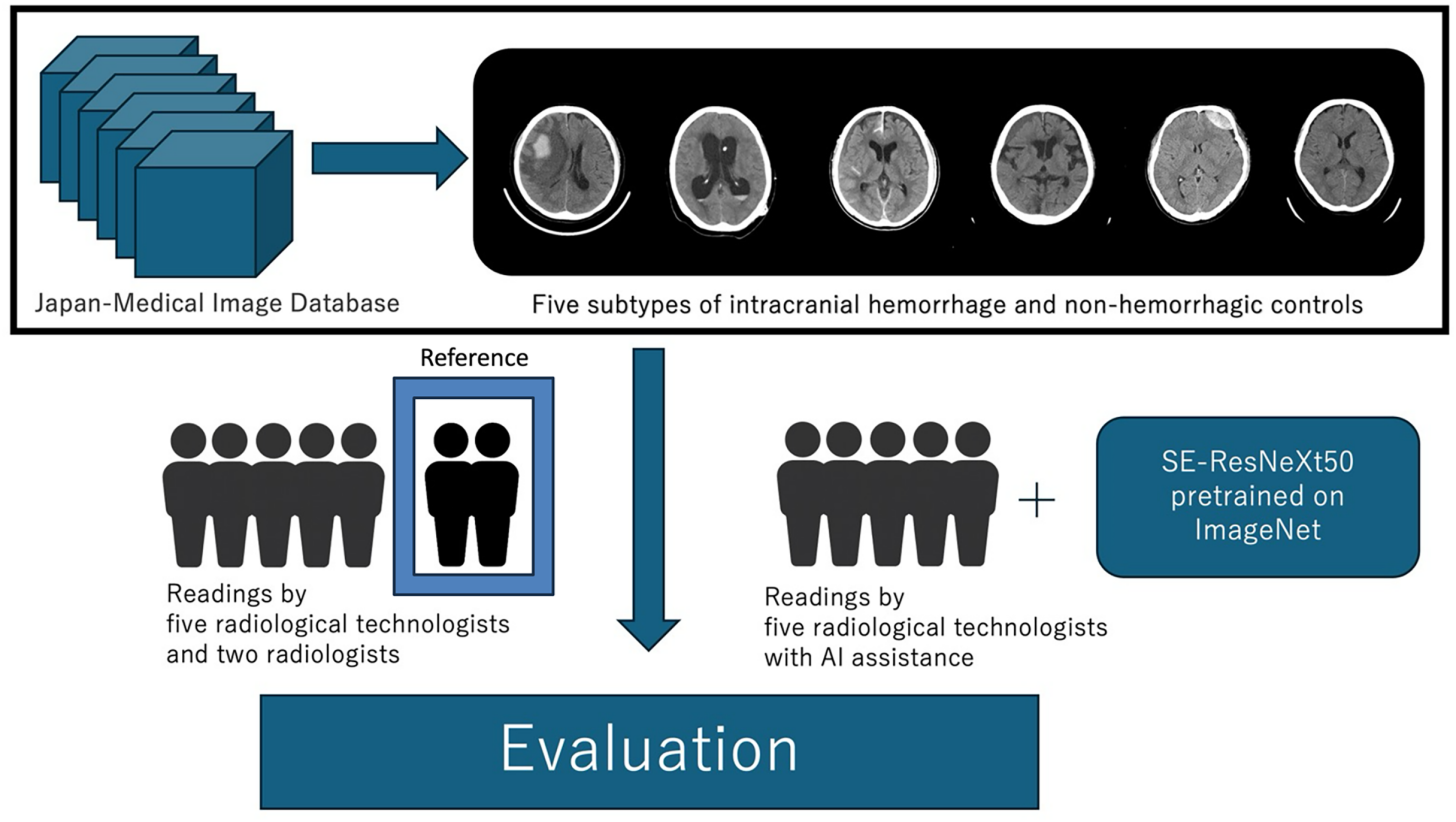
Workflow for evaluating the diagnostic performance for intracranial hemorrhage with and without AI assistance.

The case order was randomized to mitigate order effects. The scenarios (a) and (b) were separated by a 3-week washout period. The readers scored the likelihood of hemorrhage on a 10-point ordinal scale from 0 to 9. The scores from the five RTs and two radiologists were dichotomized based on a prespecified threshold (0 to 4 and 5 to 9 indicated negative and positive results, respectively) for the calculation of performance metrics such as sensitivity and specificity. The AI outputs and the radiologist and RT readings for the 100 cases were evaluated per examination. The workflow is provided in Fig. 2.

### Evaluation metrics

For each hemorrhage category, we computed the area under the receiver operating characteristic curve (ROC-AUC), accuracy, sensitivity, and specificity. The metrics for ANY were calculated at the patient level for the RSNA test set (*n* = 2,214) and the JMID test set (*n* = 2,426). The slice-level metrics for ANY were also calculated for the RSNA dataset. The patient-level metrics for ANY and the five subtypes were determined based on the 100-case JMID subset.

### Statistical analysis

Statistical analyses were performed using EZR and R. The DeLong test (DeLong, DeLong & Clarke-Pearson, 1988) was used to assess differences in the ROC**-**AUCs for the RT readings with and without AI assistance. Bonferroni correction was applied to control the family-wise error rate, and statistical significance was set to *p* < 0.00833.

## Results

### Standalone AI performance

For the RSNA public dataset (*n* = 2,214), the patient-level performance metrics for ANY were as follows: AUC, 0.978; accuracy, 0.944; sensitivity, 0.933; and specificity, 0.951. The slice level metrics were as follows: AUC, 0.9797; accuracy, 0.9336; sensitivity, 0.9299; and specificity, 0.9343.

For the JMID dataset (*n* = 2,426), the patient-level performance metrics for ANY were as follows: AUC, 0.9698; accuracy, 0.9122; sensitivity, 0.9093; and specificity, 0.9447.

For the subset of JMID used for the reader study (*n* = 100), the patient-level performance metrics for ANY were as follows: AUC, 0.999; accuracy, 0.990; sensitivity, 1.000; and specificity, 0.980.

These values of the evaluation metrics were computed using thresholds optimized based on the Youden index.

### Reader study

The reader results for the 100 JMID cases are provided in Tables 3–7. These tables also include the standalone AI and radiologist performance for comparison.

**Table 3:** Results of ROC-AUC for 100 cases in JMID.

Reader	ICH	IVH	SAH	SDH	EDH	ANY
Technologist A	0.953	0.890	0.862	0.803	0.992	0.989
Technologist A with AI	0.981	0.928	0.880	0.914	0.993	0.990
Technologist B	0.927	0.725	0.795	0.875	0.885	0.978
Technologist B with AI	0.971	0.925	0.884	0.932	0.899	0.986
Technologist C	0.895	0.763	0.878	0.760	0.733	0.964
Technologist C with AI	0.966	0.979	0.929	0.947	0.843	1.000
Technologist D	0.885	0.836	0.902	0.820	0.973	0.973
Technologist D with AI	0.983	0.942	0.948	0.985	0.943	0.987
Technologist E	0.895	0.757	0.812	0.841	0.872	0.969
Technologist E with AI	0.975	0.985	0.923	0.983	0.943	0.970
Radiologist A	0.928	0.972	0.932	0.986	0.991	0.989
Radiologist B	0.949	0.975	0.947	0.982	0.942	0.990
AI-only	0.993	0.989	0.954	0.966	0.973	0.999

**Note:**

ICH, intraparenchymal hemorrhage; IVH, intraventricular hemorrhage; SAH, subarachnoid hemorrhage; SDH, subdural hemorrhage; EDH, epidural hemorrhage (EDH); ANY, at least one subtype positive (ANY).

**Table 4:** Results of accuracy for 100 cases in JMID.

Reader	ICH	IVH	SAH	SDH	EDH	ANY
Technologist A	0.910	0.930	0.930	0.920	0.980	0.980
Technologist A with AI	0.920	0.930	0.930	0.940	0.980	0.990
Technologist B	0.870	0.890	0.910	0.910	0.950	0.920
Technologist B with AI	0.920	0.970	0.940	0.910	0.970	0.950
Technologist C	0.920	0.870	0.900	0.900	0.920	0.930
Technologist C with AI	0.940	0.950	0.910	0.940	0.940	0.990
Technologist D	0.830	0.910	0.890	0.890	0.950	0.920
Technologist D with AI	0.910	0.940	0.920	0.960	0.960	0.970
Technologist E	0.870	0.870	0.890	0.920	0.930	0.960
Technologist E with AI	0.920	0.940	0.940	0.920	0.950	0.970
Radiologist A	0.930	0.950	0.900	0.940	0.970	0.960
Radiologist B	0.920	0.950	0.890	0.920	0.950	0.980
AI-only	0.940	0.940	0.950	0.900	0.950	0.990

**Table 5:** Results of sensitivity for 100 cases in JMID.

Reader	ICH	IVH	SAH	SDH	EDH	ANY
Technologist A	0.958	0.800	0.727	0.647	1.000	0.980
Technologist A with AI	0.958	0.900	0.727	0.882	1.000	0.980
Technologist B	0.875	0.450	0.591	0.765	0.800	0.920
Technologist B with AI	0.958	0.850	0.773	0.882	0.800	0.980
Technologist C	0.750	0.450	0.682	0.471	0.500	0.920
Technologist C with AI	0.917	0.900	0.773	0.765	0.500	0.980
Technologist D	0.792	0.600	0.545	0.529	0.900	0.980
Technologist D with AI	0.958	0.750	0.773	0.941	0.700	0.980
Technologist E	0.833	0.550	0.591	0.706	0.700	0.940
Technologist E with AI	0.958	1.000	0.864	1.000	0.800	0.940
Radiologist A	0.875	1.000	0.864	1.000	0.900	1.000
Radiologist B	0.912	1.000	0.909	1.000	0.700	1.000
AI-only	1.000	1.000	0.864	1.000	0.900	1.000

**Note:**

ICH, intraparenchymal hemorrhage; IVH, intraventricular hemorrhage; SAH, subarachnoid hemorrhage; SDH, subdural hemorrhage; EDH, epidural hemorrhage (EDH); ANY, at least one subtype positive (ANY).

**Table 6:** Results of specificity for 100 cases in JMID.

Reader	ICH	IVH	SAH	SDH	EDH	ANY
Technologist A	0.895	0.962	0.987	0.976	0.978	0.980
Technologist A with AI	0.908	0.938	0.987	0.952	0.978	1.000
Technologist B	0.868	1.000	1.000	0.940	0.967	0.920
Technologist B with AI	0.908	1.000	0.987	0.916	0.989	0.920
Technologist C	0.974	0.975	0.962	0.988	0.967	0.940
Technologist C with AI	0.947	0.962	0.949	0.976	0.989	1.000
Technologist D	0.842	0.988	0.987	0.964	0.956	0.860
Technologist D with AI	0.895	0.988	0.962	0.964	0.989	0.960
Technologist E	0.882	0.950	0.974	0.964	0.956	0.980
Technologist E with AI	0.908	0.925	0.962	0.904	0.967	1.000
Radiologist A	0.947	0.938	0.910	0.928	0.978	0.920
Radiologist B	0.921	0.938	0.885	0.904	0.978	0.960
AI-only	0.921	0.925	0.974	0.880	0.956	0.980

**Table 7:** *P*-values comparing ROC-AUC without *vs*. with AI assistance.

Reader	ICH	IVH	SAH	SDH	EDH	ANY
Technologist A	0.2476	0.2993	0.4534	0.0365	0.4152	0.9669
Technologist B	0.1844	0.0004	0.0346	0.1714	0.1525	0.5535
Technologist C	0.0562	0.0001	0.2805	0.0022	0.2701	0.0689
Technologist D	0.0388	0.0071	0.3201	0.0071	0.5748	0.4895
Technologist E	0.0479	0.0120	0.0162	0.0120	0.4338	0.9457

**Note:**

ICH, intraparenchymal hemorrhage; IVH, intraventricular hemorrhage; SAH, subarachnoid hemorrhage; SDH, subdural hemorrhage; EDH, epidural hemorrhage (EDH); ANY, at least one subtype positive (ANY).

### Presence of hemorrhage (ANY)

The AUCs for the five RT interpretations without and with AI assistance ranged from 0.964 to 0.989 and from 0.970 to 1.000, respectively. The differences were not significant. The sensitivity remained in the range of 0.920–0.980 for both settings. The specificity increased from 0.860–0.980 to 0.920–1.000, and the accuracy increased from 0.920–0.980 to 0.970–0.990.

### Intraparenchymal hemorrhage (ICH)

The AUC increased from 0.885–0.953 to 0.966–0.983. Sensitivity increased from 0.750–0.958 to 0.915–0.958, and specificity changed from 0.842–0.974 to 0.895–0.948. Accuracy improved from 0.830–0.910 to 0.910–0.940.

### IVH

The AUC markedly improved from 0.725–0.890 to 0.925–0.985, representing the largest improvement among the 30 reader–task comparisons. The sensitivity increased from 0.450–0.800 to 0.750–1.00, indicating that AI markedly enhanced the detection of small or low-contrast hemorrhages. The specificity changed from 0.938–1.00 to 0.925–1.00. The accuracy increased from 0.870–0.930 to 0.930–0.970.

### SAH

The AUC increased from 0.795–0.902 to 0.880–0.948. Sensitivity improved from 0.545–0.727 to 0.727–0.864, and specificity changed from 0.962–1.00 to 0.949–0.987. Accuracy increased from 0.890–0.930 to 0.910–0.940.

### Subdural hemorrhage (SDH)

The AUC increased from 0.760–0.875 to 0.914–0.985. The sensitivity improved from 0.471–0.765 to 0.765–1.00, indicating enhanced detection of small hemorrhage with AI assistance. The specificity changed from 0.940–0.988 to 0.916–0.976. The accuracy increased from 0.890–0.920 to 0.910–0.960.

### EDH

The AUC changed from 0.733–0.992 to 0.843–0.993. The sensitivity ranged from 0.500 to 1.00. The specificity increased from 0.956–0.978 to 0.967–0.989. The accuracy changed from 0.920–0.980 to 0.940–0.980.

**Figure 3:**
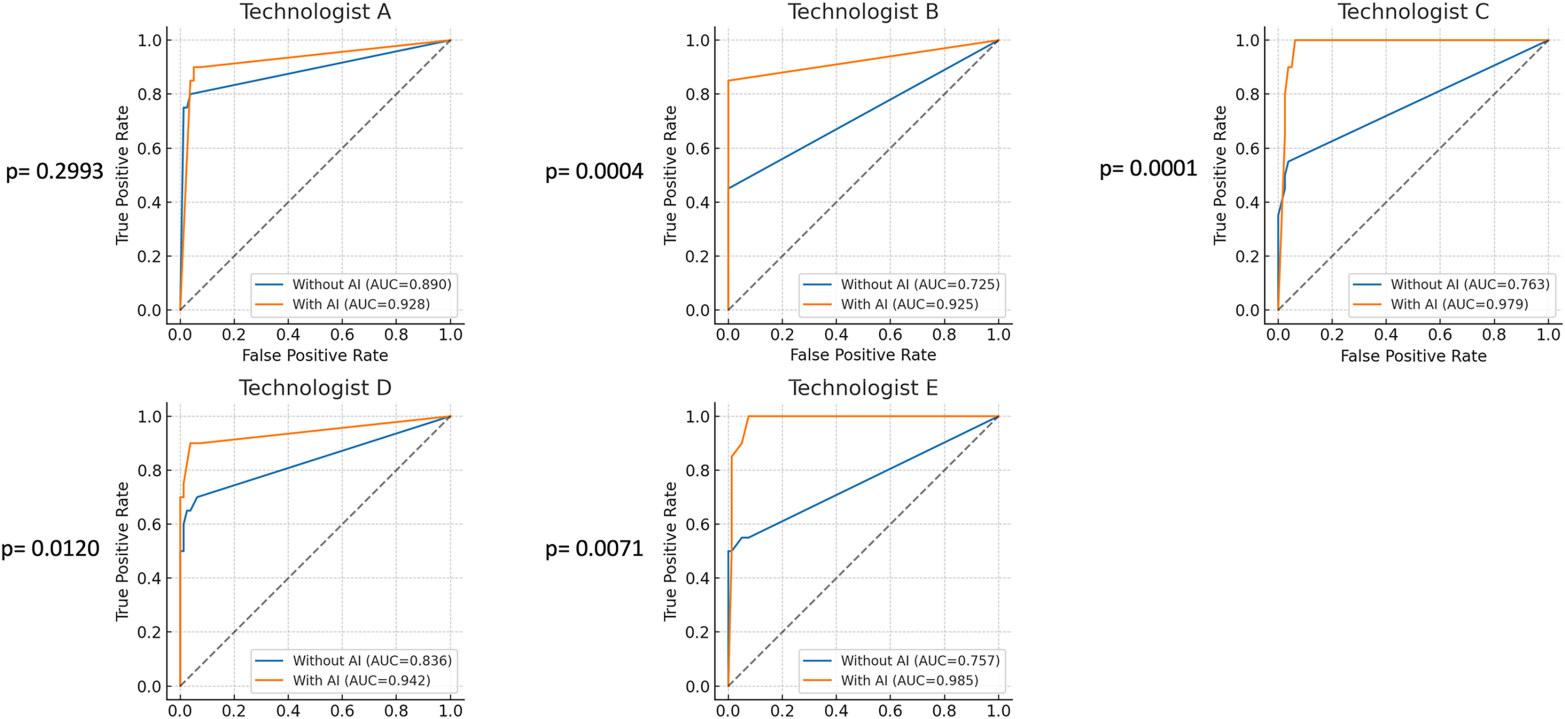
AUC comparison for IVH detection with and without AI assistance.

DeLong tests revealed significant AUC improvements with AI for five of the 30 (17%) comparisons (five RTs × five subtypes plus ANY) after Bonferroni correction (*p* < 0.00833). The largest improvement was observed for IVH detection by Reader C; the AUC increased from 0.763 to 0.979 (*p* = 0.0001). The *p*-values are provided in Table 7, and the ROC curves for IVH based on the five RTs are provided in Fig. 3.

## Discussion

We investigated the effect of AI assistance on the interpretive performance of RTs based on the findings of non-contrast head CT. First, the AUC for detecting any hemorrhage (ANY) did not differ significantly. Second, some significant AUC improvements were observed for the detection of IVH and SDH by the RTs. These findings highlight the dependence of the benefits of AI assistance on hemorrhage subtype and reader proficiency and their prominence in challenging classification scenarios.

The lack of improvement in AUC for ANY likely reflects the already high detection rates for unassisted readers, which leaves limited headroom for improvement (Bark et al., 2024; Yun et al., 2023). Determining the presence or absence of hemorrhage is more common than detailed subtyping in routine clinical workflows. This may also have contributed to the high baseline performance.

The improvement in the metrics for IVH detection was particularly marked for subtype classification. IVH were easily missed by unaided readers. Small volumes of hemorrhage may mix with cerebrospinal fluid and appear low-contrast. Their conspicuity can also vary with head position and window width/level settings (Chan, 2007). In contrast, no significant improvements in the AUC were observed for the detections of SAH or ICH. This is likely due to the high baseline discrimination for these conditions. The unclear effect of EDH may be attributable to its lower prevalence and greater variability in shape and location.

Variations in the performance of standalone AI across datasets may be attributable to differences in acquisition protocols, CT scanner characteristics, and disease prevalence. It is inappropriate to transplant cutoffs derived elsewhere without adaptation, as optimal decision thresholds are population-dependent. They should be calibrated based on the local patient populations and operational goals. They should also be reviewed periodically after deployment, and minor adjustments should be made as needed.

We compared the AUCs using the DeLong test with Bonferroni correction to control the family-wise error rate. This conservative approach enhances confidence in the observed differences. The significant improvement of AUCs for IVH and SDH are therefore likely to be robust and clinically meaningful.

The AI system used in this study incorporates a 2.5-D input (adding the immediately adjacent superior and inferior slices to the target slice) and applies three window settings (brain, subdural, and bone) simultaneously. This approach accentuates subtle gray-level differences and enhances the conspicuity of small hemorrhages (Chang et al., 2018; Nguyen et al., 2020; Karki et al., 2020; Burduja, Ionescu & Verga, 2020; Daugaard Jørgensen et al., 2022). It may have increased the sensitivity for cases likely to be missed, as reflected by the higher AUCs. The slice-level results for the RSNA dataset demonstrate superior accuracy of our single AI model relative to those reported in previous study (Wang et al., 2021).

This study has limitations. First, the reader set comprised 100 cases balanced for positive and negative findings may not reflect real-world prevalence and may reduce the external validity of the study. Second, five RTs with 5–12 years of experience participated. Caution is warranted when generalizing the results to RTs with other experience levels. Third, we did not evaluate operational outcomes such as reading time, time to urgent communication, susceptibility to automation bias, or ease of explanation. To prevent radiographers from blindly trusting incorrect AI outputs, it is crucial to provide education that helps them understand the trends demonstrated in this study, ensuring that the AI is utilized as a tool to complement human judgment. Fourth, site-specific verification and calibration using local data are required to disentangle the effect of dataset heterogeneity on the performance of AI (McKinney et al., 2020).

## Conclusions

AI assistance may help stabilize RT-led Preliminary Clinical Evaluation or Preliminary Image Evaluation and triage during periods of limited radiologist coverage, in non-specialist settings, and under high workload when decision-making may degrade or communication errors may occur (Alexander-Bates et al., 2021). Positioning AI software as a targeted support tool (Topol, 2019) and coupling it with focused case-based training may help reduce the frequency of missed detections for difficult subtypes such as IVH and some cases of SDH. AI is effective as an assistive technology, but it should be used to complement, not replace, human readers by addressing specific weaknesses. This approach is more likely to result in reliable and clinically useful improvements in practice (Angkurawaranon et al., 2023).

## Supplemental Information

10.7717/peerj.21414/supp-1 Supplemental Information 1 Python code for AI. Differences between the Python code used in this study and that used in the previous study.

10.7717/peerj.21414/supp-2 Supplemental Information 2 Raw data (raw results) obtained from radiologists, technologists, and AI. Data used for the statistical analyses performed in this study.

10.7717/peerj.21414/supp-3 Supplemental Information 3 CT scan parameters for the 100 cases used in the reader study.

10.7717/peerj.21414/supp-4 Supplemental Information 4 Representative cases with CT images and detailed clinical descriptions. Representative cases demonstrating the added value of the AI system, as well as its limitations.

## Additional Information and Declarations

### Data Availability

The following information was supplied regarding data availability:

The data and code are available in the Supplemental Files.
