# Supplementary material for: Development and evaluation of a deep learning-assisted diagnostic support system for radiographer preliminary clinical evaluation of intracranial hemorrhage
Source: PeerJ. 2026 Jun 17;14:e21414. doi: 10.7717/peerj.21414 (PMC13282943; doi:10.7717/peerj.21414)

**Supplementary Material: Representative cases**

This section provides representative cases demonstrating the added value of the AI system, as well as its limitations.

A. Added Value of AI (Diagnostic Improvement)

The following examples illustrate cases where radiographers initially missed the hemorrhage but successfully corrected their diagnosis after reviewing the AI predictions.

Case A1: Intraventricular Hemorrhage (IVH)

- Diagnostic shift: 0 out of 5 radiographers detected IVH without AI → All 5 radiographers correctly detected it with AI.
- Clinical findings: Right cerebellar hemorrhage. The ruptured hematoma demonstrates extension into the subarachnoid space and the ventricular system.


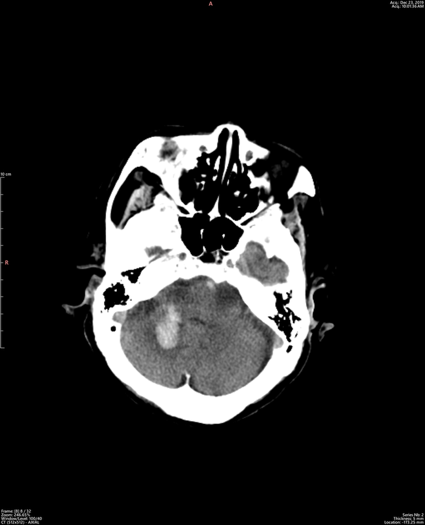

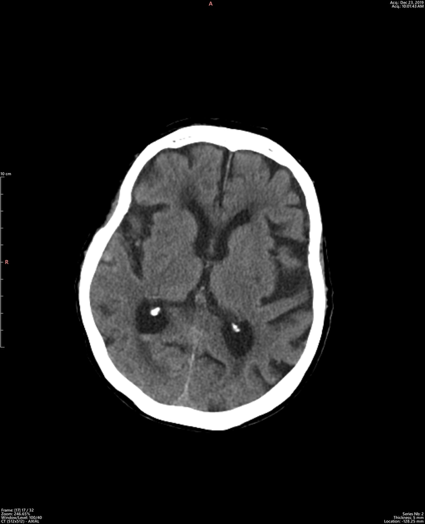

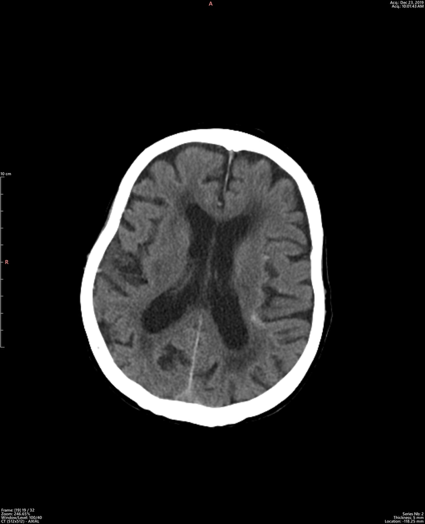


Case A2: Intraparenchymal Hemorrhage (ICH)

- Diagnostic shift: 1 out of 5 radiographers detected ICH without AI → 4 radiographers correctly detected it with AI.
- Clinical findings: Right temporal epidural hematoma. The accompanying ICH shows a tendency to expand.


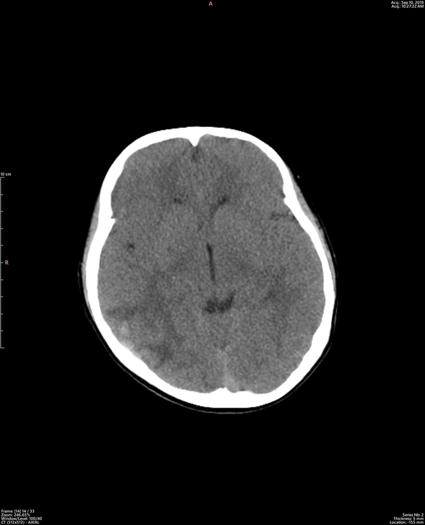

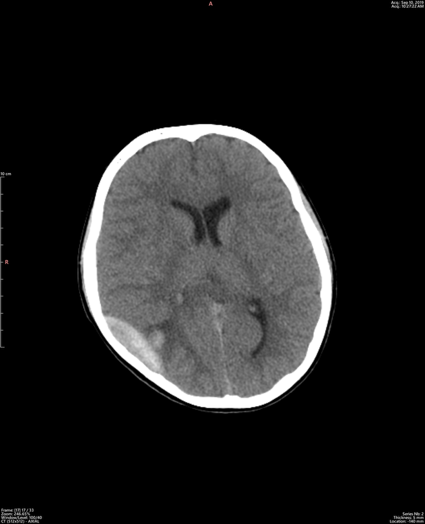

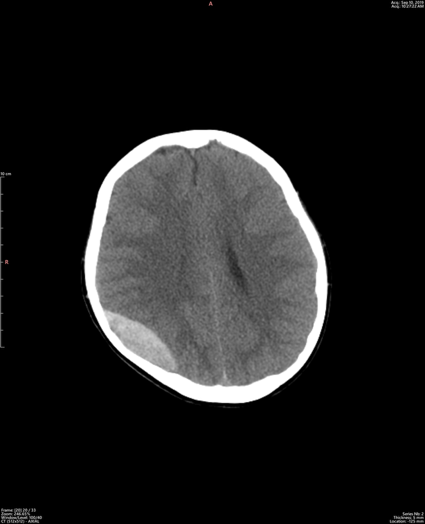


Case A3: Subarachnoid Hemorrhage (SAH)

- Diagnostic shift: 0 out of 5 radiographers detected SAH without AI → All 5 radiographers correctly detected it with AI.
- Clinical findings: Extensive subcortical hematoma extends from the left frontal to the parietal lobe. SAH on the surface of the right cerebral hemisphere has become apparent.


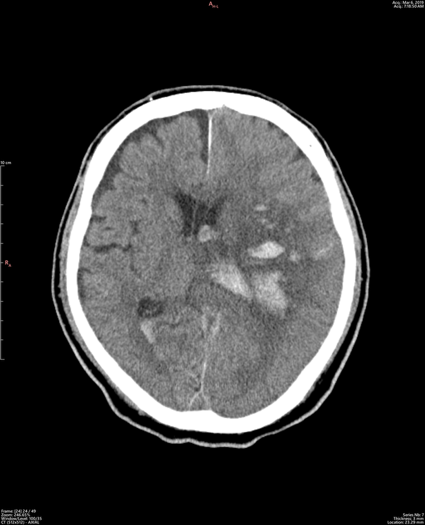

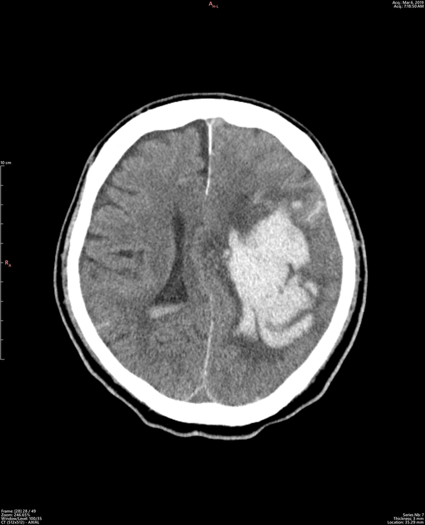

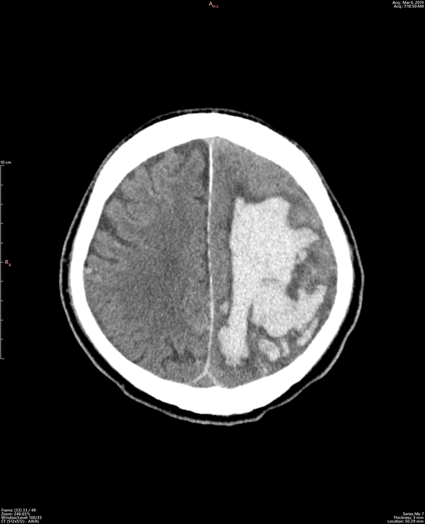


B. Limitations of AI (Missed Detections)

The following example illustrates a challenging case where the AI system failed to detect a hemorrhage (false negative). The radiographers also failed to identify the hemorrhage with and without AI assistance.

Case B1: Subarachnoid Hemorrhage (SAH)

- No diagnostic shift: SAH was missed by all 5 radiographers with and without AI assistance.
- Clinical findings:
  - Bilateral subdural hematomas are present.
  - A hematoma associated with a contusion is noted at the base of the left frontal lobe.
  - SAH is scattered within the cerebral sulci.


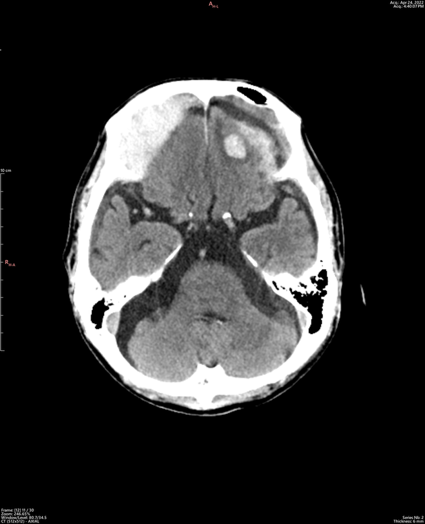

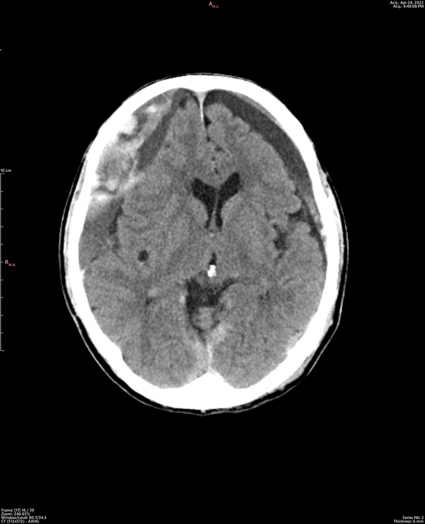

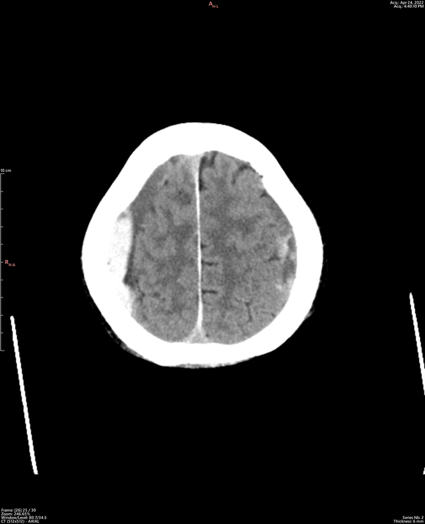

Supplement: Supplemental Information 4 — Representative cases demonstrating the added value of the AI system, as well as its limitations. [file peerj-14-21414-s004.docx]
